# Evaluation of complex congenital heart disease and associated complications in newborns, infants and small children using multi-detector CT

**DOI:** 10.1186/1532-429X-11-S1-P67

**Published:** 2009-01-28

**Authors:** Joachim G Eichhorn, Sebastian Ley, Christian Fink, John Cheatham, Wim Helbing, Frederick Long

**Affiliations:** 1 University Children's Hospital, Pediatric Cardiology, Heidelberg, Germany; 2 University Children's Hospital, Pediatric Radiology, Heidelberg, Germany; 3 German Cancer Research Center (DKFZ), Radiology, Heidelberg, Germany; 4 Nationwide Children's Hospital, Pediatric Cardiology, Columbus, OH USA; 5 Erasmus MC, Sophia University Children's Hospital, Pediatric Cardiology, Rotterdam, Netherlands; 6 Nationwide Children's Hospital, Pediatric Radiology, Columbus, OH USA

**Keywords:** Congenital Heart Disease, Vascular Anomaly, Complex Congenital Heart Disease, Arterial Ring, Minimize Radiation Exposure

## Introduction

Multi-detector CT (MDCT) with multiplanar and three-dimensional (3D) reconstruction has become an important first-line imaging tool in diagnosis of congenital vascular anomalies (VA) and airway diseases in children.

## Purpose

To assess the diagnostic value of MDCT for evaluation of VA and associated complications in newborns and infants.

## Methods

At 4 centers, 262 children (mean age: 7 ± 7 months, range: 6 hours to 24 months) were examed. The diagnoses included: suspected of having in-stent stenosis after stent placement to treat vascular stenoses (n = 72), pulmonary atresia (32) and stenosis (48) with collateral arteries, arterial rings and slings (28), aortic arch anomalies (15), bronchoscopy revealed stenosis(23), abnormal pulmonary venous return (15) and others (29). The CT exams were performed on varying scanners (4 up to 64 slices, collimation isotrope 0.4–1.25 mm; scan-time 2–20 s, usage of low dose protocols: 80–120 kVp, 60–80 mA) under controlled ventilation or free breathing, mostly without ECG-gating to minimize radiation exposure. The image quality was rated using a 5-point score. Image findings were correlated to echocardiography, conventional digital catheter angiography (DA), bronchoscopy, and intraoperativ findings. The effects of dose on image quality were also evaluated, retrospectively.

## Results

**Figure 1:**
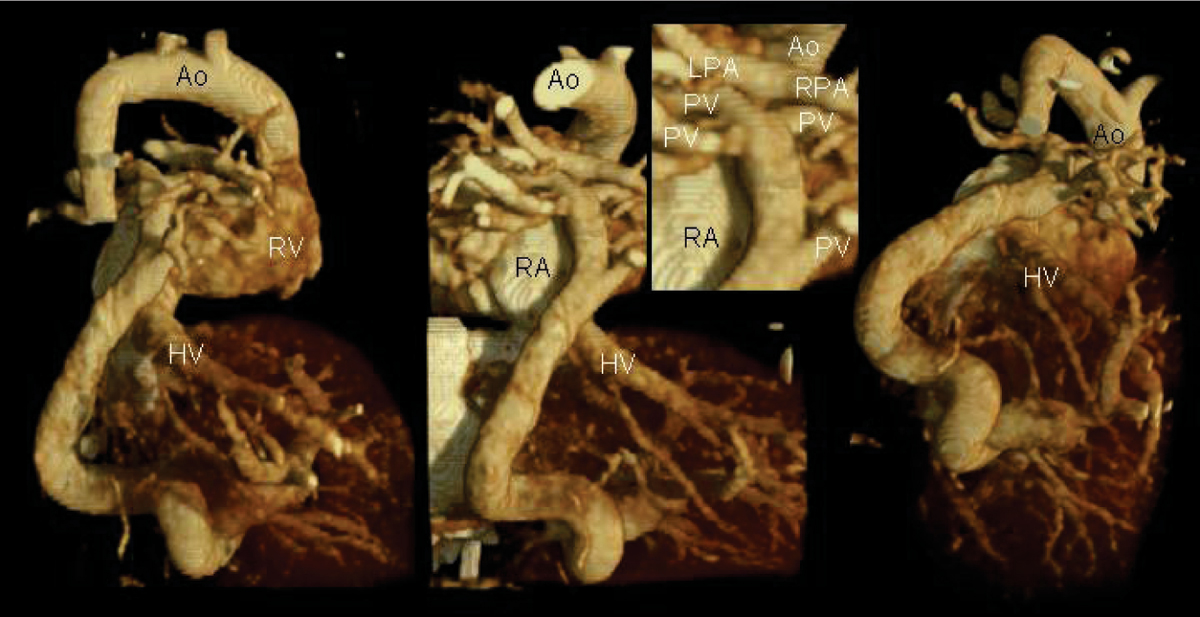
**Newborn (body weight 3 kg) with heterotaxia, tetralogy of Fallot with pulmonary atresia and total anomalous pulmonary venous drainage to the portal vein**. 3D volume rendered images obtained on a 64 slice MDCT scanner following hand injection of contrast in the umbilical venous catheter. Images show the pulmonary veins (PV, diameter < 1 mm) draining to a confluens which crosses the diaphragm supplying the hepar veins (HV) in posteriorlateral and inferior projections. Aorta (Ao), right atrium and ventricel (RA and RV), left and right pulmonary artery (LPA and RPA).

High quality MDCT data were almost free of cardiac and respiratory motion. Images were scored for vascular contrast and for delineation of the tracheobronchial tree in >95% of all cases as excellent or good, showing a significant improving with increasing number of detectors. Significant differences between higher and lower radiation settings were not found. The radiation exposure was mostly less than 2 mSv (range 1.1–3.2). High radiation exposure settings did not improve image quality. VA morphology and topography in relation to adjacent structures, e.g. tracheal and esophageal compression caused by an arterial ring or sling, could be assessed exactly and allowed the final diagnosis. Even smallest vessels with a diameter of less than 1 mm (see figure 1), could be identified and excellently visualized. Eighty-two percent (215/262) of all patients had benefited from MDCT: Digital angiography was neither necessary to perform surgical or interventional planning nor to exclude a VA, or radiation doses and sedation time due to interventional procedures could be reduced markedly.

## Conclusion

A 3D submillimeter evaluation of the heart and great vessels can be achieved routinely in a matter of seconds with little motion artifacts, without general anesthesia and with much less radiation exposure than previously thought. Nevertheless, the optimal radiation dose settings for performing cardiac CT in infants and pediatric patients are still being worked out. The use of CT for screening may result in a net decrease in overall radiation decreasing the number of diagnostic cardiac catherizations. CT can now be regarded as the modality of choice as a minimally invasive, robust, and accurate technique for the diagnosis of complex VA even in the group of newborns and infants or critically ill patients. Its accuracy for detecting VA appears at least equivalent to catherizations while it is more accurate in delineating potential life-threatening complications. This advance should have the greatest impact in the smallest, youngest, and most critically ill children with congenital heart disease.

